# Reflections on the Treatment of Insanity

**Published:** 1819-02

**Authors:** George Nesse Hill


### *For the London Medical and Physical Journal.*

### *Reflections on the Treatment of Insanity;* by GEORGE NESSE HILL, Esq.

AMONG some very excellent reflections on the highly important subject of blood-letting in maniacal disease, by your learned and valuable correspondent Dr. Kinglake, inserted in your Number for November, p. 372, he very pertinently observes—"My motive for submitting the above observations to the public is to restrain what would appear to be a very pernicious practice,—that of carrying bleeding to a most daring and hazardous extent in affections of the brain, however circumstanced:" justly adding—"It seems, therefore, that the present licentious abuse of bleeding requires that the experienced and the reflecting part of the medical faculty should enter their protest against the immoderate length to which it is often carried."

If Dr. K. had done me the honour of perusing the "Essay on the Prevention and Cure of Insanity," published in the year 1814, he would have found the following observations bearing directly upon the interesting point on which he has so ably descanted, and confirming his appropriate remarks. Such readers of your valuable Journal into whose hands the Essay has not yet come, will not object to the citation of a few proofs that the subject in question has not been heretofore wholly neglected.

"It is much too common a practice with professional gentlemen who do not attempt, nor indeed wish, to take farther steps in the cure of insanity, to instantaneously direct a copious bleeding of the maniacal delirious subject. This practice is as indiscriminate as it is frequent, and meriting high reprobation: notwithstanding such a procedure has the sanction of ages and of names illustrious in the public opinion, still it is injuriously erroneous."
*Essay on Prev. and Cure of Insan.* 285.

"No sooner is the existing disease determined to belong to this class [mania], or indeed lunacy in any shape, but bleeding, hellebore, chains, painful degrading coercion, starving, and dark dungeons, crowd on the mind."—"The evacuation of blood, however, takes the lead, dangerous as the operation must ever be when practised upon high maniacal subjects; and, when it fails in reducing the raging, miserable, and loathed sufferer to calmness, it is again and again repeated. '*Iterare pugnam.*' '*Saignez le encore;*' as though it was the *sinè quâ non*, as indeed it generally is of folly and ignorance, daily adding more incurable subjects to the enormous number already shut out from society."—He was bled thirteen times, every time till he fainted, in the compass of six days: he recovered, but soon relapsed."—"I have known another case, where it was supposed the patient was bled to death, without its producing any alteration in the complaint." *Domestic Guide*, p. 105. Dr. Ferriar *on the Conversion of Diseases*, p. 136.

*Essay*, p. 286.

"It is an undoubted truth that, in fifty maniacs, labouring under the highest degree of the sthenic form, not more than from seven to ten of them will require this most powerful means of reduction of the vital power; and let it never be forgotten, that sudden and profuse bleeding is always (even in this form, however furious,) highly dangerous, and *never* necessary."

*Ibid*, p. 288.

"In fact, to quote M. Pinel's own words, the subject has been 'thrown into a kind of imbecility and idiotism by the excessive use of the lancet.' *Pinel*, p. 88. 'Nay, immediate death has been the result.' See Whytt *on Nerv. Dis.* p. 590. Let, then, the young practitioner read such a case seriously, and pause before he adds to the number of incurable lunatics."

*Ibid*, p. 290.

"Blood is, in high sthenic insanity, always what is called buffy; but such an appearance by no means indicates the necessity of repeating the evacuation until the buffiness ceases to exist; although such a practice has been often recommended, and there is no doubt been faithfully executed, till stopped by the departure of the sufferer 'to realms unknown.'"

*Ibid*, p. 290.

—But your Journal must not be loaded with useless repetition, however interested every human being that breathes is in the subject: sufficient has been recorded to prove that a solemn "protest against the immoderate length to which bleeding is often carried" has long since been entered. Yet, notwithstanding this fact, such able men as Dr. Kinglake cannot too frequently second the efforts of less popular writers to stem the torrent of folly, remove the films of ignorance, and rouse the attention of the careless and the indifferent,—not to mention the blameably mischievous, or the wilfully indifferent.

At this momentous crisis, it would be almost an unpardonable violation of feeling to close this letter without remarking that eventful time has again disclosed to our pained senses a striking and, if we choose it, a salutary, though severe, lesson on the tremendous consequences of defective energy in the prompt and effectual treatment of nascent, but decisive, mental aberration, and the ultimate prevention of suicide. Shall the shock which public feeling has recently sustained be merged in the stream of common events, and merely serve to "point a moral or adorn a tale?" or shall it be improved to the benefit of the present rising generation? Among the medical faculty, there will not surely be a solitary instance of one member so lost to the honour and the glory of his profession, as not to rouse himself to the determination that no part of the circle which his individual practice embraces shall want the timely scrutiny so eminently calculated to prevent the repetition of events so deplorable, and, when committed, so uselessly regretted.

A subject of greater moment cannot occupy the attention of your readers. Impressed with this conviction, it is neither arrogance nor presumption to remind such of them as have perused the work already quoted, of the doctrines and plan of treatment laid down in the chapter on the Prevention of Insanity:* a very cursory re-perusal will convince them that, had the principles there inculcated been faithfully acted upon, the last great man, whose loss we now lament, would, in all human probability, still have breathed the "vital air."

*Chester; Dec.* 1, 1818.

* The highly-intelligent minds of the two last exalted beings, which were gradually undermined by disease overwhelming the intellectual powers, enabled the sufferers to describe the gradual but steady approaches of insanity, coinciding most minutely with the general history given in the Essay. Of this assertion every reader must be convinced, who will be at the trouble of comparing the evidence given on the coroner's inquest and the sentiments of the author.

